# Robust Light State by Quantum Phase Transition in Non-Hermitian Optical Materials

**DOI:** 10.1038/srep17022

**Published:** 2015-11-23

**Authors:** Han Zhao, Stefano Longhi, Liang Feng

**Affiliations:** 1 Department of Electrical Engineering, The State University of New York at Buffalo, Buffalo, NY 14260, USA; 2 Dipartimento di Fisica, Politecnico di Milano and Istituto di Fotonica e Nanotecnologie del Consiglio Nazionale delle Ricerche, Piazza L. da Vinci 32, Milano I-20133, Italy

## Abstract

Robust light transport is the heart of optical information processing, leading to the search for robust light states by topological engineering of material properties. Here, it is shown that quantum phase transition, rather than topology, can be strategically exploited to design a novel robust light state. We consider an interface between parity-time (PT) symmetric media with different quantum phases and use complex Berry phase to reveal the associated quantum phase transition and topological nature. While the system possesses the same topological order within different quantum phases, phase transition from PT symmetry to PT breaking across the interface in the synthetic non-Hermitian metamaterial system facilitates novel interface states, which are robust against a variety of gain/loss perturbations and topological impurities and disorder. The discovery of the robust light state by quantum phase transition may promise fault-tolerant light transport in optical communications and computing.

Metamaterials have offered a new paradigm of designing unprecedented material properties, revolutionizing our fundamental understanding in optics^1^. While in the past the design of metamaterials was mainly focused on the real permittivity-permeability plane, the emergence of parity-time (PT) symmetry^2^ for non-Hermitian Hamiltonians in quantum field theory has been guiding the studies of metamaterials into the entire dielectric permittivity plane with a delicate interplay of index, gain and loss^3^,^4^,^5^,^6^. In spite of non-Hermiticity, completely real eigen spectra, corresponding to PT symmetric phase, can still be expected. By increasing the gain/loss contrast, the eigen spectra become complex and the system can transit into PT broken phase^3^,^4^,^5^,^6^. Recent investigations of PT symmetric metamaterials have enabled a variety of intriguing optical phenomena, including effective manipulation of cavity oscillating modes^7^,^8^,^9^ and unidirectional light transport^10^,^11^,^12^,^13^,^14^,^15^,^16^, which promise new functionalities for integrated photonics information processing.

To secure fault-tolerant light transport for optical communications, robust optical interface states, inspired by topological insulators and quantum Hall systems in condensed-matter physics, have been developed^17^,^18^,^19^,^20^,^21^,^22^,^23^. From a topological perspective, a system can acquire a non-negligible geometric phase (also called Berry phase) under a cyclic and adiabatic variation in a parameter space, which classifies the topological orders of matter^24^. An important signature of topological effects is the presence of topological states at the interface between two media with different topological orders. Because of the persistent Berry phase underlying each topological order, the interface states are topologically protected against local perturbations to the interface. In optics, topological light states are immune to backscattering of light^25^, promising applications in optical information processing^26^. Nevertheless, these robust light states are strongly dependent of stringent topological designs, which are difficult to achieve using the low-accuracy but cost-effective fabrication technologies^27^ especially in high-density photonics integration, hindering their application feasibilities. Such strong topological dependence also circumscribes the position of the interface and thus limits the freedom of further light manipulation. Hence, it is important to search for other robust mechanisms that are free of topological orders and capable of relocating the interface on-demand.

In this Letter, we suggest that, rather than topology, quantum phase transition in PT metamaterials can be exploited to realize robust light states that can be controlled at will. While the interplay between quantum phase transition and the topological nature associated with PT metamaterials is rather challenging and remains an open question, we explore the Berry phase^28^,^29^,^30^,^31^, extended to the complex domain in a one-dimensional (1D) PT symmetric Su-Schrieffer-Heeger (SSH) model^32^, to analyze and reveal quantum phase transitions under the same topology. Remarkably, we predict, for the first time, the existence of robust light states across the boundary between two media with unbroken and broken PT symmetry. Beyond their topological counterpart, light states induced by quantum phase transition turn out to be robust against global topological impurities and disorders even away from the interface.

The 1D PT symmetric SSH model consists of periodic coupled dimers with onsite gain and loss [Fig. 1(a)]. The coupling strength between adjacent dimers can be controlled by the distance between them and their gain/loss contrast. Using the tight-binding approximation, coupled mode equations read


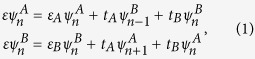


where *t*_*A*_ and *t*_*B*_, called hopping amplitudes, are interdimer and intradimer coupling coefficients, respectively, *ε* denotes the eigen energy of the system, and *ε*_*A*_ and *ε*_*B*_ are onsite energies, satisfying the condition of 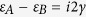, where *γ* is the gain/loss amplitude. The corresponding non-Hermitian Hamiltonian is


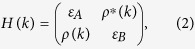


where 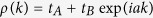, *a* is the periodicity and *k* is the quasi-momentum in the Brillouin zone. Assuming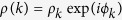, the energy dispersion can be obtained:


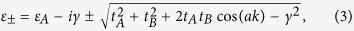


corresponding to a two-level system. The associated right and left eigenvectors in dual space are,


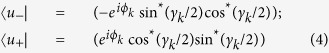


and


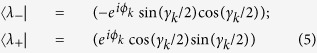


where 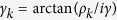. Due to intrinsic non-Hermiticity, 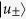, the right eigenvector of 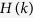, does not generally equal to the eigenvector 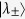 of 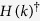. Therefore, eigenvectors in non-Hermitian systems are non-orthogonal, making the Berry connection 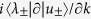 complex rather than real as in Hermitian cases. In a 1D lattice, the geometric phase is acquired when the quasi momentum sweeps a closed loop of the Brillouin zone^33^. Thus, Berry phases of the upper and lower bands are


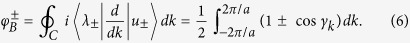


Figure 1(b,c) show the calculated complex Berry phase of the PT symmetric SSH model under the condition of 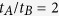. If the onsite gain/loss vanishes, the geometric phase is quantized to be either 0 or π, depending on the ratio of hopping amplitudes 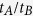: 0 if 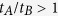 and π if 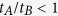, corresponding to an accumulated geometric phase arising in a periodic structure when the Bloch quasi-momentum spans the Brillouin zone^34^,^35^,^36^. However, in the presence of onsite gain/loss, the system is non-Hermitian, such that the associated Berry phase of each individual band becomes complex in general. In contrast to the quantized geometric phase in Hermitian systems, Berry phase continuously varies by increasing the onsite gain/loss contrast. Such a continuous variation of the complex Berry phase arises from the mode non-orthogonality of non-Hermitian systems. Moreover, the complex Berry phase spectra can be applied to rigorously analyze the quantum phase transition. Any abrupt transition or diverging point denotes the transition threshold where the system-associated quantum phase transits from one to another. Note that quantum phase transition here refers to an abrupt change of ground-state Berry phase when the parameter of the Hamiltonian is varied. Different from the terminology in condensed matter physics, thermodynamic issues do not apply to the discussed optical settings. It is clear that three different quantum phases are observed as a function of onsite gain/loss: *Phase I:* PT symmetric phase across the entire Brillouin zone; *Phase II:* Mixture of PT symmetry and PT breaking; and *Phase III:* PT breaking across the entire Brillouin zone, which is consistent with the direct analysis of energy dispersions (see Supplementary Material). While the energy eigen spectra of Phase I is completely real, the corresponding Berry phase is complex, even though the system stays in PT symmetric phase. The observed complex Berry phase is directly related to Wannier-Stark ladders with complex energies in the presence of an external force^37^.

The topological nature of the system is determined by the global Berry phase, corresponding to the summation of Berry phase in both lower and upper bands^28^. While Berry phase of each individual band continuously varies in different quantum phases, the global Berry phase remains quantized independent of onsite gain/loss, demonstrating the same topological nature of the system regardless of quantum phase transition. From the relation of ground-state Berry phase, a unique genus by quantum phase invariant may exist in non-Hermitian systems, leading to a novel robust interface state if transiting from complete PT symmetry (Phase I with gain/loss amplitude *γ*_1_) to PT breaking (Phase III with gain/loss amplitude *γ*_2_). This interface between different quantum phases can be manipulated by controlling the gain/loss contrast with the selective pumping strategie^38^, exhibiting great freedom and tenability compared to topological photonics. The general solution of the interface state is


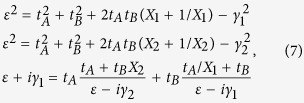


where 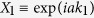 and 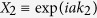, and 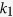 and 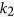 are quasi momenta in each sub-lattice, respectively, and *ε* is the energy of the interface state (see Supplementary Material). Based upon the numerical solution of Eq. (7), a bound state that exponentially decays away from the interface exists under the condition that


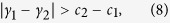


where 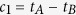 and 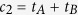 are the two critical points for quantum phase transitions. The demonstrated interface state becomes robust with its eigen energy remaining unperturbed against the variation of onsite gain/loss, if


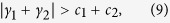


which ensures that the interface state energy stays unchanged at the middle of the band gap of the Phase I lattice. Hence, while Eq. (8) shows the condition of the existence of the bound state that can take place without quantum phase transition (see Supplementary Material), it suggests, together with Eq. (9), that the robust light state can only occur if quantum phase transits across the interface from Phase I to Phase III. Also notice that the condition of the bound state set by Eq. (8) is only valid when at least one semi-lattice is within Phase I.

Figure 2(a) shows a realistic scheme to mimic the SSH model using arrays of coupled waveguides, where onsite gain/loss coefficients are represented by the imaginary part of the indices of the waveguides. The interdimer and intradimer coupling coefficients are effectively controlled by the distances between adjacent waveguides (see Supplementary Material)^22^,^23^. Such couplings are then periodically repeated along the whole system, such that the entire system is of the same topological nature by which topological phase transition is prohibited. It is clearly demonstrated that two lattices under the same topological order successfully induce the bound interface state due to quantum phase transition from PT symmetry (Phase I) to PT breaking (Phase III) with field confinement at the interface and asymmetric exponential decays in two semi-lattices [Fig. 2(b)]. In contrast to the common optical localized state that locates within the band gap of the systems on both sides, this interface state can fall within the continuum. This is because the energy spectrum is complex and the interface state is amplified at a higher rate than the Bloch states supported in both semi-space lattices. Remarkably, as suggested by Eq. (9),the interface state by Phase I and Phase III is robust and its energy almost remains the same with the same field distribution profile regardless of the onsite gain/loss variation of the waveguide lattice. Although the robust interface state only exists between Phase I and Phase III, a bound state can emerge if the gain/loss contrast on two sides is large enough, satisfying Eq. (8). In this case, the bound state can be observed between different quantum phases [see Fig. 2(c,d)] or under the same quantum phase (see Supplementary Material). However, these bound states are not robust and their energy is sensitive to onsite gain/loss perturbation (see Supplementary Materials for more details). Compared to the topological interface state realized by controlling topological orders of two semi-lattices, i.e. the ratios of interdimer and intradimer hoping amplitudes, the robust interface state by the quantum phase transition is to manipulate the non-Hermiticity of two PT semi-lattices to simultaneously satisfy Eqs. (8) and (9).

To validate the robustness of quantum phase transition induced interface state, we intentionally introduce the perturbations of hopping amplitudes into the system. For example, an impurity is created by shifting the first A dimer in the right lattice [Fig. 3(a)], such that the couplings become 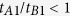 locally while global coupling remains the same (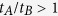). Remarkably, in spite of a less than 1% frequency change, the interface state persists with field concentrated at the same site, suggesting its robustness against local topological impurities. This is different than a defect introduced in the topologically induced interface state where the same topological impurity on hopping amplitudes would simply shift the most concentrated field to the next site, i.e. the second A site in our case. To further test the robustness against the global topological variation, the ratio of the hopping amplitudes of the right sub-lattice is tuned from 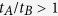 to 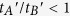 [Fig. 3(b)]. Because of the introduced topological operation, the corresponding topological order of the right sub-lattice varies. Intuitively, topological phase transition is created across the interface, leading to the topologically induced field concentration at the first B site of the left sub-lattice. However, the quantum phase transition induced interface state still remains with almost no frequency change and its field is most concentrated at the first A site of the right sub-lattice, evidently demonstrating that the interface state by quantum phase transition is robust against global topological disorders. This topology-insensitive property overcomes the limitation of the stringent topological dependence in topological photonics where topological disorder may destroy the topological phase transition and thus ruin its induced interface state^39^.

While the demonstrated robust interface state is well investigated based upon the tight-binding SSH model, we conjecture that such a robust light state is rather general in non-Hermitian PT symmetric systems. Another example is the scenario of the interface composed by two continuously modulated PT symmetric lattices: 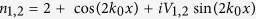, where 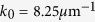, as shown in Fig. 4(a). While it is difficult to directly calculate Berry phase associated with continuous PT modulation, similar quantum phase transition can be well controlled by varying the imaginary index modulation (i.e. *V*_1_ or *V*_2_). Two critical points, 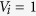 and 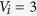, divide the whole spectrum into the same 3 quantum phases: Phase I, Phase II, and Phase III. The result shown in Fig. 4(b) is consistent with what we observed with the tight-binding SSH model. With the same topological nature across the interface, the phase transition from unbroken to completely broken PT symmetry induces the interface state with strong field localization at 194.4 THz. The same as the result in Fig. 2(b), this interface state is robust and its energy is immune against the variation of the gain/loss contrast in sub-lattices.

In conclusion, we discovered a novel robust light state that arises from quantum phase transition in PT symmetric systems. The complex Berry phase in non-Hermitian systems can be used as an order parameter to characterize the associated quantum phase transition. Based on the study of the Berry phase in the PT symmetric SSH model, a novel interface state induced by quantum phase transition is demonstrated. Complementary to topological photonics, the demonstrated interface state by quantum phase transition is more robust against global topological impurities and may be flexibly manipulated. While our results are based on one-dimensional models and the corresponding interface states are static, the concept of the interface state by PT phase transition can be extended to higher dimensions^40^, where robust propagative edge states sustained by quantum phase transitions can arise.

## Additional Information

**How to cite this article**: Zhao, H. *et al.* Robust Light State by Quantum Phase Transition in Non-Hermitian Optical Materials. *Sci. Rep.* **5**, 17022; doi: 10.1038/srep17022 (2015).

## Supplementary Material

Supplementary Materials

## Figures and Tables

**Figure 1:**
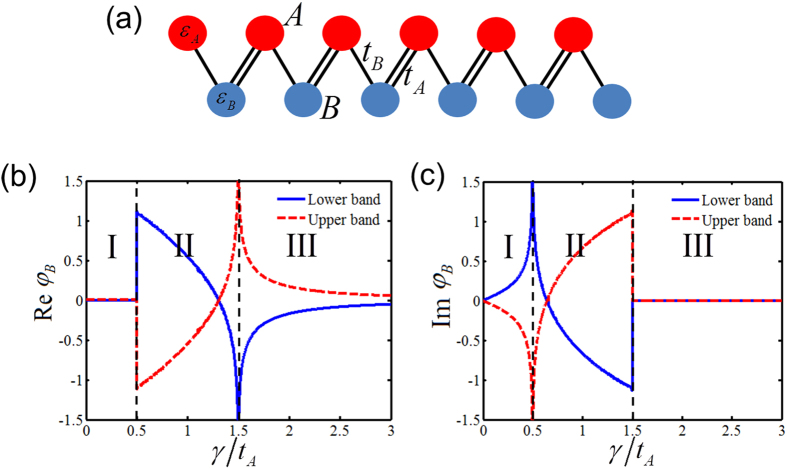
Complex Berry phase. (**a**) Schematic of the PT symmetric SSH model, where all the A sites are gain and B sites are loss. Each individual site is dominantly coupled with the two nearest neighboring sites with interdimer and intradimer coupling strengths, *t*_*A*_ and *t*_*B*_, respectively. (**b**) and (**c**) are the real and imaginary ground state Berry phase spectra for 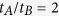, respectively, with increasing onsite gain/loss, showing two critical points at 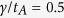 and 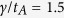. These two critical points correspond to the quantum phase transition points and divide the spectra into three quantum phases: *Phase I*: PT symmetric phase; *Phase II*: partially broken-PT phase; and *Phase III*: completely broken PT phase. The global Berry phase, the summation of individual Berry phase in both upper and lower bands, remains unchanged regardless of onsite gain/loss, manifesting the same topological nature of different quantum phases.

**Figure 2:**
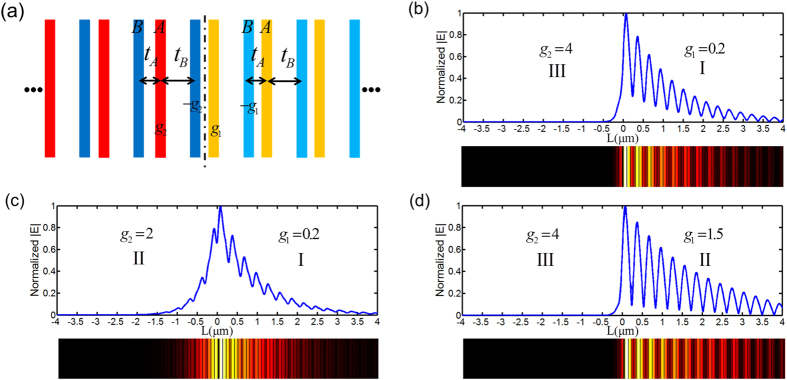
Interface states by non-Hermiticity. (**a**) Schematic of the PT symmetric SSH model and its interface state in optical settings. A/B sites are replaced with 88 nm-wide gain/loss waveguides of the index 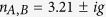, respectively. The interdimer distance is 122 nm, and the intradimer distance is 176 nm (center to center), making approximately 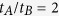. The hopping amplitudes remain the same in the whole system to avoid topological phase transition. In this configuration, the real part of the effective refractive index for waveguides is 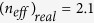 and the imaginary part depends on the parameter 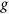 with an approximate relation of 
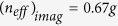
. The two critical points referring to quantum phase transitions are 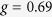 and 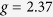. (**b**) Eigen mode simulation of the interface state by Phase I and Phase III at 194.4 THz, where 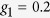 (right sub-lattice) and 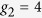 (left sub-lattice). (**c**) The interface state by Phase I and Phase II at 216.0 THz, where 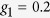 (right) and 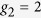 (left). (**d**) The interface state by Phase II and Phase III at 185.1 THz, where 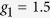 (right) and 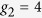 (left). While different interface states are demonstrated, only in (**b**) the eigen energy remains the same as 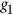 and 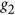 are perturbed.

**Figure 3:**
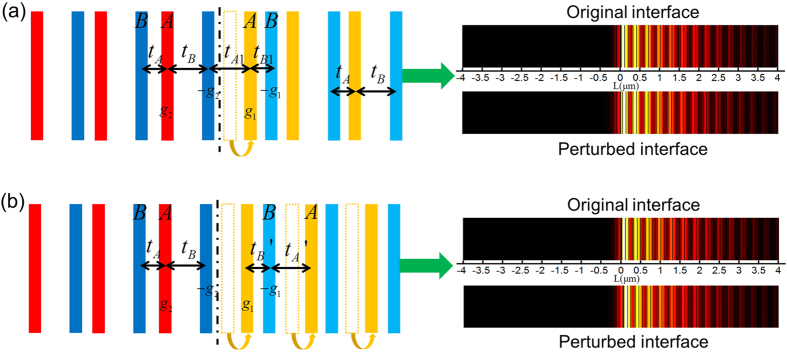
Robustness of the interface state. In both (**a**) and (**b**), the original interface state by Phase I (right sub-lattice) and Phase III (left sub-lattice) is plotted for a comparison. (**a**) A local topological impurity next to the interface is introduced by displacing the first A site in the right sub-lattice, where the local coupling strength changes from 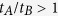 to 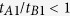. Compared to its original at 194.4 THz, the interface state is well preserved with a slight frequency change to 193.1 THz. (**b**) A global topological disorder is introduced in the right sub-lattice, where all the A sites shift to the right such that the hopping amplitudes change from 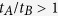 to 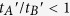. The interface state remains almost at the same frequency (194.8 THz) with the introduced topological perturbation, showing consistent field distributions.

**Figure 4:**
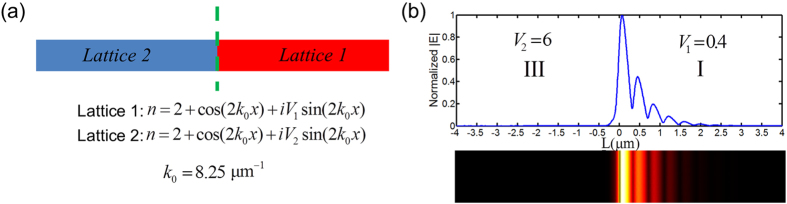
Interface state by continuously modulated PT photonic lattices. (**a**) Schematic of the interface formed by two PT symmetric lattices, Lattice 1 (right): 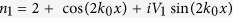, and Lattice 2 (left): 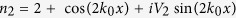, where 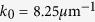 and the corresponding quantum phases can be controlled by *V*_1_ and *V*_2_. The two critical phase transition points are 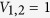 and 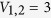. The real part modulations are identical for both semi-infinite lattices, ensuring the same topological order. (**b**) Interface state by Phase III and Phase I at 194.4 THz when 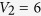 and 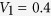, respectively. This interface state also resides at the middle of the band gap of Lattice 1 even if *V*_1_ and *V*_2_ are perturbed, showing its robustness immune to the gain/loss variation in two sub-lattices.
