# Supplementary material for: Robust Light State by Quantum Phase Transition in Non-Hermitian Optical Materials
Source: Sci Rep. 2015 Nov 23;5:17022. doi: 10.1038/srep17022 (PMC4655477; doi:10.1038/srep17022)
Supplement: Supplementary Materials [file srep17022-s1.pdf]

Supplementary Material

Robust Light State by Quantum Phase Transition in Non-Hermitian Optical Materials

Han Zhao[1], Stefano Longhi[2] and Liang Feng[1]

[1]*Department of Electrical Engineering, The State University of New York at Buffalo, Buffalo, NY 14260, USA*

[2]*Dipartimento di Fisica, Politecnico di Milano and Istituto di Fotonica e Nanotecnologie del Consiglio Nazionale delle Ricerche, Piazza L. da Vinci 32, Milano I-20133, Italy*

Dispersion relation of PT symmetric SSH model

The quantum phase transition revealed by the complex Berry phase can be validated by direct inspection of the energy spectrum. The band structure of the PT symmetric SSH model is plotted in Fig. S1 for increasing values of loss/gain parameter. The PT symmetric phase corresponds to a completely real energy spectrum, as shown in Figs. S1(a) and S1(d). This is Phase I where PT symmetry is preserved across the Brillouin zone. If the onsite gain and loss coefficients are increased till the critical point of $\gamma/t_A = 0.5$, the bands coalesce at the boundary of the Brillouin zone and complex-conjugate energies appear. This accidental degenerate point moves towards the center of the Brillouin zone if the gain and loss coefficients are further increased. In this case, the system falls into Phase II where broken PT symmetry occurs near the boundary of the Brillouin zone and PT symmetry still remains around the center, as shown in Figs. S1(b) and S1(e). With further increasing gain and loss till $\gamma/t_A > 1.5$, the system transits into Phase III with energy spectrum becoming completely complex. The bifurcation happens in the imaginary spectrum with a pair

of complex conjugate eigenvalues, denoting broken PT symmetry across the entire Brillouin zone, as shown in Figs. S1(c) and S1(f). The two phase transition points, $\gamma/t_A = 0.5$ and $\gamma/t_A = 1.5$, are well mapped with the discontinuous variation points in the complex ground-state Berry phase spectra. This is because the Berry phase offers an effective measure to the order parameter for quantum phase transition in a quantum system.

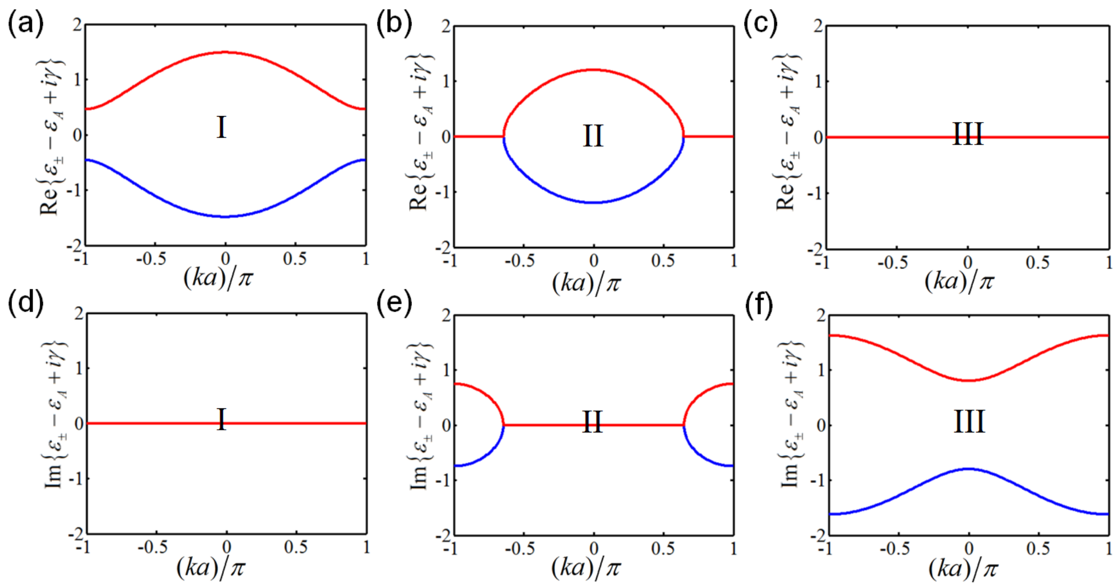

Fig. S1. Band structures of different quantum phases: (a) and (d): Phase I; (b) and (e): Phase II, and (c) and (f): Phase III, where red and blue curves denote the upper band and the lower band, respectively. The upper panels are the real eigen spectra while the lower panels are imaginary. Parameters of the SSH model in these plots are: $t_A/t_B = 2$. γ is varied so that the system can be tuned into different quantum phases.

Semi-analytical solution of interface state in PT symmetric SSH model

Here, we consider a PT-SSH based interface structure as depicted in Fig. S2.

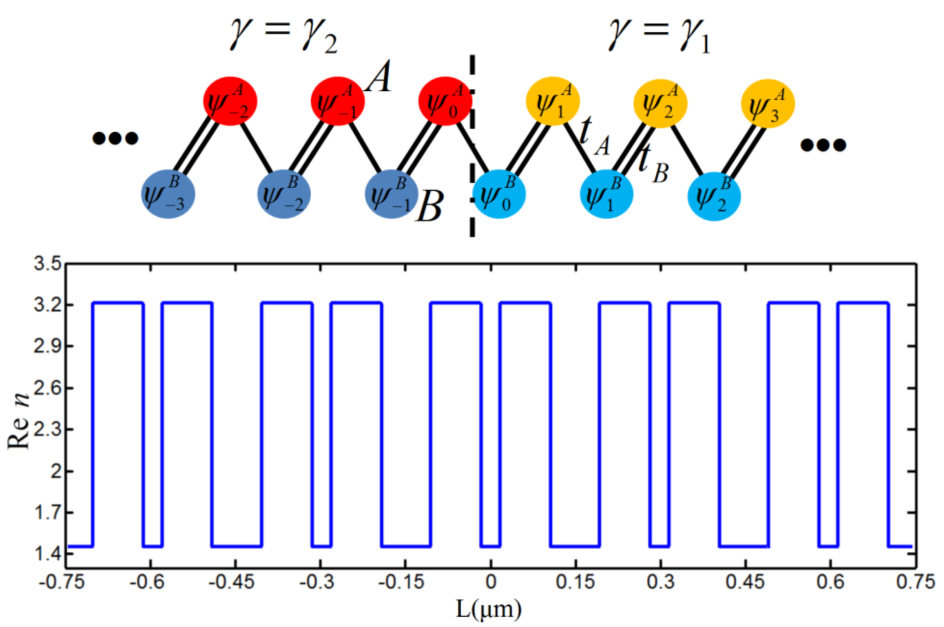

Fig. S2. Schematic of interface structure of SSH model (upper panel) and the real part index profile of the waveguide array to mimic the SSH model (lower panel). Two semi-lattices are of different onsite gain and loss coefficients γ_1 and γ_2, respectively, while maintaining the same topological order.

For simplicity, we assume $\varepsilon_A = -\varepsilon_B = i\gamma$ on both sides. The coupled mode equations are

$$\varepsilon\psi_n^A = i\gamma_2\psi_n^A + t_A\psi_n^B + t_B\psi_{n-1}^B, \quad n \le 0, \tag{S1}$$

$$\varepsilon\psi_n^B = -i\gamma_2\psi_n^B + t_A\psi_n^A + t_B\psi_{n+1}^A, \quad n \le -1, \tag{S2}$$

$$\varepsilon\psi_n^A = i\gamma_1\psi_n^A + t_A\psi_n^B + t_B\psi_{n-1}^B, \quad n \ge 1, \tag{S3}$$

$$\varepsilon\psi_n^B = -i\gamma_1\psi_n^B + t_A\psi_n^A + t_B\psi_{n+1}^A, \quad n \ge 0. \tag{S4}$$

Then the interface state solution, if it exists, should be in the form of

$$\psi_n^A = \begin{cases} A_1 \exp(-iq_1 n), n \ge 1 \\ A_2 \exp(-iq_2 n), n \le 0 \end{cases}, \tag{S5}$$

$$\psi_n^B = \begin{cases} B_1 \exp(-iq_1 n), n \ge 0 \\ B_2 \exp(-iq_2 n), n \le -1 \end{cases}, \tag{S6}$$

where q_1 and q_2 are complex Bloch wave numbers in two sub-lattices. For bound

interface states, it is required that $\mathrm{Im}(q_1) < 0$ and $\mathrm{Im}(q_2) > 0$. From Eq. (S1-S6), the energy ε of interface state satisfies

$$\varepsilon^2 = t_A^2 + t_B^2 + 2t_A t_B \cos q_1 - \gamma_1^2, \tag{S7}$$

$$\varepsilon^2 = t_A^2 + t_B^2 + 2t_A t_B \cos q_2 - \gamma_2^2. \tag{S8}$$

To find the relations among A_1, A_2, B_1 and B_2, we set $n=0$, $n=-1$ and $n=1$, respectively, which yields

$$(\varepsilon - i\gamma_1)A_1 = t_A B_1 + t_B e^{iq_1} B_1, \tag{S9}$$

$$(\varepsilon - i\gamma_2)A_2 = t_A B_1 + t_B e^{iq_2} B_2, \tag{S10}$$

$$(\varepsilon + i\gamma_1)B_1 = t_A A_2 + t_B e^{-iq_1} A_1, \tag{S11}$$

$$(\varepsilon + i\gamma_2)B_2 = t_A A_2 + t_B e^{-iq_2} A_2. \tag{S12}$$

From Eqs. (S8), (S10) and (S12), one can find $B_1 = B_2$. Let $B_1 = B_2 = B$, then A_1 and A_2 are derived as

$$\begin{cases} A_1 = \dfrac{t_A + t_B e^{iq_1}}{\varepsilon - i\gamma_1} B \\ A_2 = \dfrac{t_A + t_B e^{iq_2}}{\varepsilon - i\gamma_2} B \end{cases}. \tag{S13}$$

Thus, after the introduction of two quantities $X_1 \equiv \exp(iq_1)$ and $X_2 \equiv \exp(iq_2)$, Eqs. (S7), (S8) and (S11) form algebraic equations

$$\begin{aligned} \varepsilon^2 &= t_A^2 + t_B^2 + 2t_A t_B (X_1 + 1/X_1) - \gamma_1^2 \\ \varepsilon^2 &= t_A^2 + t_B^2 + 2t_A t_B (X_2 + 1/X_2) - \gamma_2^2, \\ \varepsilon + i\gamma_1 &= t_A \frac{t_A + t_B X_2}{\varepsilon - i\gamma_2} + t_B \frac{t_A / X_1 + t_B}{\varepsilon - i\gamma_1} \end{aligned} \tag{S14}$$

where X_1, X_2 and ε can be obtained. Thus, if there exists a solution with $|X_1| > 1$ and $|X_2| < 1$, the amplitude of onsite field exponentially fades away from the interface and the bound interface state can be found at the energy ε.

Numerical solutions of Eq. (S14) reveal that there is at least one interface solution if the right lattice is in Phase I and the difference of the gain/loss coefficients is larger

than the difference between two critical points driving quantum phase transitions. Moreover, $\mathrm{Re}\left[\varepsilon-\left(\varepsilon_A-i\gamma\right)\right]$ stays at 0 if the summation of onsite gain/loss on both sides exceeds the summation of two critical points. These calculations establish Eqs. (8) and (9) given in the main text.

Waveguide array implementation of the SSH model

We implemented the non-Hermitian tight-binding SSH model by considering light propagation in an array of evanescently coupled optical waveguides with tailored guide separation and gain/loss terms. A schematic of the waveguide array is shown in Fig.S2, lower panel. It consists of a sequence of step-index waveguides with 88 nm channel width, alternating separation 122 nm and 176 nm, and with the refractive index profile (real part) shown in the figure (the refractive index of 3.21 and the step index variation of 1.76). Gain and absorption are phenomenologically introduced by adding an imaginary part to the refractive index.

Provided that each waveguide is weakly coupled by the two adjacent waveguides with coupling efficiencies κ_A and κ_B, the coupled waveguide array can be modeled by the coupled mode theory, which reads

$$\begin{cases} -i\dfrac{d}{dz}A_j=\beta_A A_j+\kappa_A B_{j-1}+\kappa_B B_j \\ -i\dfrac{d}{dz}B_j=\beta_B B_j+\kappa_A A_{j+1}+\kappa_B A_j \end{cases}. \tag{S15}$$

where A_j and B_j are the amplitudes trapped in the *j*th A and B sites, and β_A and β_B are the propagation constants of gain and loss waveguides, respectively. The coupling efficiencies for two straight waveguides without gain and loss can be numerically calculated by

$$\kappa=\frac{k_0^2}{2k_1}\frac{\int(\varepsilon_W-\varepsilon_S)E_1^*\cdot E_2 ds}{\int E_1^*\cdot E_1 ds}. \tag{S16}$$

where E_1 and E_2 are transverse field distribution of fundamental modes in the two

waveguides, and ε_W and ε_S are dielectric constants of the waveguide and background, respectively, while k_0 and k_1 are propagation constants in vacuum and waveguide, respectively. For gain/loss balanced PT-symmetric waveguide arrays, the detuning can also be estimated by

$$\beta_A - \beta_B = i2\alpha = i2n_{imag}k_0. \qquad \text{(S17)}$$

where n_{imag} is the magnitude of imaginary part of the gain/loss waveguide effective index. Parameter values entering in coupled-mode equations have been obtained from the actual index profile of Fig.S2 by means of commercial COMSOL software at the reference wavelength of around 1550 nm.

By solving linear ordinary differential equations for the amplitudes of each individual waveguide, the coupled mode theory has been demonstrated to provide an accurate solution of vectorial Maxwell equations [1]. Also notice the similarity between Eq. (S15) and Eq. (1) which describes the PT symmetric SSH model under tight-binding approximation.

Non-robust optical interface states

The solution of Eq. (S14) suggests that the bound state occurs if the onsite gain/loss coefficients in two semi-lattices, γ_1 and γ_2, respectively, are of large enough contrast (for example, as shown by Eq. (8) if one lattice is in Phase I). Therefore, onsite gain/loss variation Phase I to Phase II may also induce a bound state without satisfying Eq. (9), as numerically validated in Fig. S3(a) [i.e. Fig. 2(c)]. However, the real part of the energy here varies if the gain/loss coefficients of two lattices change within the corresponding ranges of their associated quantum phases (Phase I and Phase II). Meanwhile, Phase II and Phase III semi-lattices can also form the bound state as shown in Fig. S3(b) [i.e. Fig. 2(d)]. The corresponding energy is located at the band merging point of the Phase II semi-lattice. However, such a bound state is not robust as well since the frequency of the band merging point of the phase II semi-lattice decreases with increasing its gain/loss coefficient.

More interestingly, the bound state can still occur though two semi-lattices are within the same quantum phase, if they are associated with large enough difference of gain/loss coefficients. Here, we performed an eigen mode simulation for the case with left and right sub-lattices both in Phase II but with opposite sign of onsite gain/loss ($g_1 = -g_2 = 1.1$, other settings are identical compared with Fig. 2). Figure S3(c) shows the simulated bound interface state at the frequency of 248.7 THz. Because of the same gain and loss amplitude on both sides of the interface, the field distribution is symmetric. However, such an interface state is also not robust, as its energy ε clearly changes with perturbations of the onsite gain/loss amplitude. Figure S3(d) shows another interface state at 216.4 THz with $g_1 = -g_2 = 2$ (that is still in Phase II). The real part of the interface state energy ε shifts down by 13%.

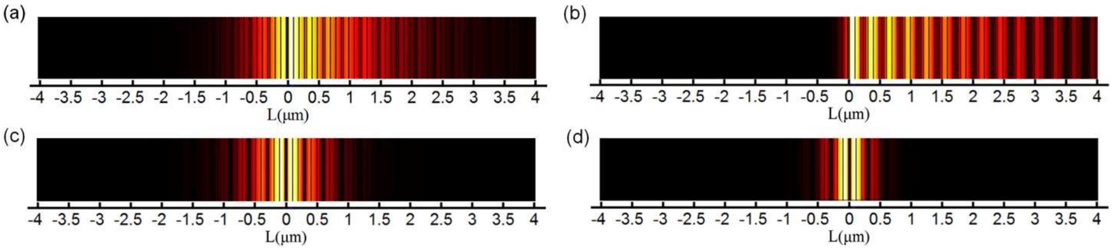

Fig. S3. Bound states that are not robust against perturbation of onsite gain/loss amplitude. The bound states (a) and (b) are formed by onsite gain/loss variation from Phase I to Phase II and from Phase II to Phase III, respectively. They are the same as the results shown in Fig. 2(c) and Fig. 2(d), respectively. (c) and (d) show the bound states by two semi-lattices with an opposite sign of gain and loss coefficients. All other settings are kept the same compared with Fig. 2 except for the onsite gain and loss coefficient: in (c), $g_1 = -g_2 = 1.1$; and in (d), $g_1 = -g_2 = 2$.

Therefore, it is evident that only the interface state by phase transition from pure PT symmetry (Phase I) to PT breaking (Phase III) satisfying both Eqs. (8) and (9) is robust similar to the topological interface state, as its energy ε remains the same against perturbations of onsite gain/loss amplitudes.

References

[1] See, for instance: J. Huang, Opt. Soc. Am. A 11, 963 (1994) and references therein.
